# Automated T-Cell Proliferation in Lab-on-Chip Devices Integrating Microfluidics and Deep Learning-Based Image Analysis for Long-Term Experiments

**DOI:** 10.3390/bios15100693

**Published:** 2025-10-13

**Authors:** María Fernanda Cadena Vizuete, Martin Condor, Dennis Raith, Avani Sapre, Marie Follo, Gina Layedra, Roland Mertelsmann, Maximiliano Perez, Betiana Lerner

**Affiliations:** 1 Mertelsmann Foundation, 79104 Freiburg, Germany; mc294@email.uni-freiburg.de (M.F.C.V.); avani@labmaite.com (A.S.); roland.mertelsmann@uniklinik-freiburg.de (R.M.); 2 Facultad de Farmacia y Bioquímica, Universidad de Buenos Aires, Ciudad Autónoma de Buenos Aires 1113, Argentina; 3 Faculty of Biology, Albert-Ludwigs-University of Freiburg, 79104 Freiburg, Germany; 4 CONICET, Instituto de Investigaciones en Microbiología y Parasitología Médica (IMPaM), Universidad de Buenos Aires, Ciudad Autónoma de Buenos Aires 1121, Argentina; 5 LABMaiTE GmbH, 79110 Freiburg, Germany; martin@labmaite.com (M.C.); dennis@labmaite.com (D.R.); 6 Neurorobotics Lab, Department of Computer Science, Albert-Ludwigs-University of Freiburg, 79104 Freiburg, Germany; 7 Department of Medicine I, Faculty of Medicine, Medical Center–University of Freiburg, 79106 Freiburg, Germany; marie.follo@uniklinik-freiburg.de; 8 Lighthouse Core Facility, Faculty of Medicine, Medical Center–University of Freiburg, 79106 Freiburg, Germany; 9 IREN Center, National Technological University, Buenos Aires 1706, Argentina; gina-layedra@hotmail.com; 10 Collaborative Research Institute Intelligent Oncology (CRIION), 79110 Freiburg, Germany; 11 Department of Electrical and Computer Engineering, Florida International University, Miami, FL 33174, USA

**Keywords:** deep learning, image analysis, microfluidics, Lab-on-chip (LOC) devices, T-cells

## Abstract

T cells play a pivotal role in cancer research, particularly in immunotherapy, which harnesses the immune system to target malignancies. However, conventional expansion methods face limitations such as high reagent consumption, contamination risks, and difficulties in maintaining suspension cells in dynamic culture environments. This study presents a microfluidic system for long-term culture of non-adherent cells, featuring automated perfusion and image acquisition. The system integrates deep learning-based image analysis, which quantifies cell coverage and estimates cell numbers, and efficiently processes large volumes of data. The performance of this deep learning approach was benchmarked against the widely used Trainable Weka Segmentation (TWS) plugin for Fiji. Additionally, two distinct lab-on-a-chip (LOC) devices were evaluated independently: the commercial ibidi^®^ LOC and a custom-made PDMS LOC. The setup supported the proliferation of Jurkat cells and primary human T cells without significant loss during medium exchange. Each platform proved suitable for long-term expansion while offering distinct advantages in terms of design, cell seeding and recovery, and reusability. This integrated approach enables extended experiments with minimal manual intervention, stable perfusion, and supports multi-reagent administration, offering a powerful platform for advancing suspension cell research in immunotherapy.

## 1. Introduction

Suspension cells, including various immune cells, such as T cells and B cells, play crucial roles across diverse fields of biological research. Unlike adherent cells, which require attachment to a surface, suspension cells remain free-floating in the growth medium, mimicking conditions found in body fluids like blood and lymph. This characteristic is especially important in cancer research, where non-adherent immune cells are central to immunotherapy research [1,2].

Immunotherapy harnesses the patient’s immune system to target and eliminate cancer cells. Among its approaches, adoptive cell therapy (ACT) has emerged as a highly promising strategy due to its personalized nature, which enhances therapeutic success. ACT involves isolating immune cells from the patient, genetically engineering them to express specific receptor proteins, expanding them ex vivo, and reinfusing them into the patient to selectively target cancer cells [2,3,4,5].

Despite its potential, ACT faces notable technical challenges during the ex vivo expansion of T cells. Traditional flask-based methods require multiple handling steps, increasing the risk of contamination. Although effective, perfusion-based systems often require substantial volumes of culture medium, sometimes exceeding 25 L, making them resource-intensive. Additionally, newer bioreactor technologies, although innovative, can unintentionally alter cellular kinetics, potentially impacting cell behavior and therapeutic efficacy [6,7,8].

Microfluidics offers a compelling solution to many of these limitations. This technology, often referred to as the “miniaturization of the laboratory,” uses devices with microscale chambers and channels to create controlled environments. These systems enable continuous perfusion of fresh cell culture media, providing nutrients while removing waste products at a regulated flow rate. Such features facilitate the establishment of long-term cultures, controlled microenvironments, and real-time analysis of the outlet medium [9,10].

Lab-on-chip (LOC) devices, a cornerstone of microfluidics, integrate multiple laboratory functions into compact platforms measuring only a few square centimeters. These devices, made from biocompatible materials such as polydimethylsiloxane (PDMS), act as microreactors that support the growth of prokaryotic and eukaryotic cells, allowing for the co-culture of different cell types. Their small size reduces the volume of samples and reagents required, while the customizable design of their microchannels allows adaptation to specific experimental objectives, including multiplexed assays. Furthermore, LOC devices enable the parallelization of experiments and reproducible results [9,10,11,12,13,14].

Although microfluidics has been widely applied to adherent cells [10,11,15], working with suspension cells presents unique challenges. Most notably, the risk of cell loss during medium exchange in long-term perfusion cultures has limited the development of automated platforms specifically tailored for ex vivo T-cell expansion [9]. To address this, many existing systems focus on short-term analysis, relying on strategies such as physical clamping that may induce mechanical stress [16] or have different goals, such as spheroid formation [17]. More sophisticated platforms exist, but they are often tailored to unrelated applications, like massive scalability for microbial systems biology [18], or involve complex fabrication that limits their accessibility [19].

Establishing long-term, image-based cultures introduces a further bottleneck: managing and analyzing the large data volumes generated. This challenge is compounded by the difficulty of processing label-free images, which are essential to preserve cellular viability. While conventional machine learning tools (e.g., the widely used Trainable WEKA Segmentation (TWS) plugin in Fiji or custom workflows in Matlab) are widely used [16,19,20], their consistency can be challenged by artifacts in dynamic culture environments. Deep learning, with architectures such as U-Net and the efficient YOLOv8, has emerged as a powerful alternative. However, its application to T-cell imaging has largely focused on cell classification in static cultures [21] or analyzing non-microscopy data from sources like flow cytometry [22]. This reveals a gap, as a systematic evaluation of these models against established baselines for quantifying T-cell proliferation in long-term LOC cultures has not been fully explored.

In this study, we address these gaps by presenting an integrated microfluidic system for the long-term cultivation of non-adherent cells, which combines automated perfusion control and image acquisition with LOC devices. Image analysis was performed using deep learning-based segmentation and benchmarked against the TWS plugin for Fiji. A central question for researchers adopting this technology is the choice between standardized commercial systems and adaptable, in-house fabricated devices; however, direct comparisons to guide this decision for T-cell expansion are lacking. To address this, two alternatives were evaluated: a commercial µ-Slide Spheroid Perfusion device (ibidi^®^, Gräfelfing, Germany; “ibidi LOC”) and a custom-fabricated PDMS device (“PDMS LOC”, IREN Center, National Technological University, Argentina), to assess their respective advantages and limitations for supporting T-cell expansion.

## 2. Materials and Methods

### 2.1. Microfluidic Setup and System Calibration

Experiments in both LOC devices were conducted using the automated Elveflow^®^ microfluidic system (Elvesys, Paris, France). This system utilizes a pressure controller (OB1) to regulate pressure-driven flow, creating a pressure difference inside the reservoir (15 mL and 50 mL Falcon^®^ tubes [Life Technologies GmbH, Darmstadt, Germany] filled with various liquids) that drives the liquid toward the outlet. By adjusting the input of gas pressure, a precise and pulseless flow rate is achieved [23].

The pressure applied to maintain a stable flow rate is based on data provided by the MFS flow sensor (Elvesys, Paris, France), which monitors flow rates ranging from 2 to 80 µL/min with an accuracy of 5% and a response time of 35 ms. To ensure flow stability with a variation of ±1 µL/min, the control parameters were optimized (P: 0.04, I: 0.01 for this specific setup). Furthermore, the MFS flow sensor detected flow irregularities, such as bubbles or clots in the tubing, ensuring the system is free of bubbles before starting experiments [23,24,25].

**Figure 1:**
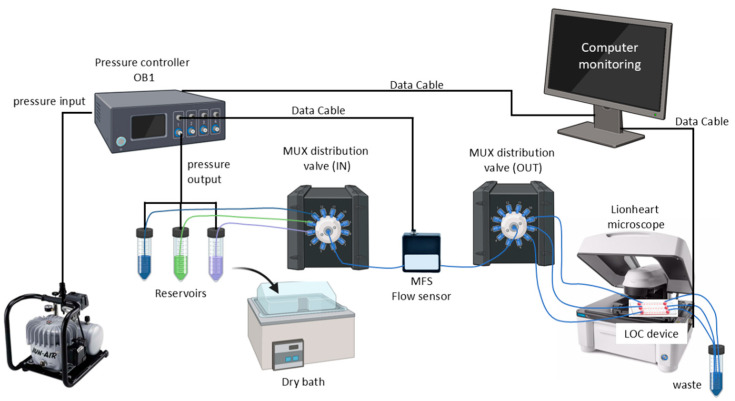
Schematic of the microfluidic system. This system uses a pressure controller (OB1, Elvesys, France) that receives pressure from an air compressor and is connected from the front side to the reservoirs (Falcon^®^ tubes of 15 mL and 50 mL filled with different liquids). Feedback is received from the MFS flow sensor, which detects the flow rate generated by the applied pressure. The liquids are transported by tubing attached to distribution valves, called MUX. Two valves were used, one to distribute the different liquids from the reservoirs and a second one to distribute them to the LOC device. The LOC device is imaged with an automated microscope. All the equipment was monitored using a computer.

The liquids were transported through tubing connected to distribution valves called MUX. For an adequate distribution of the fluids, two valves were used: one for the distribution of the different fluids from the reservoirs and a second one for the distribution to the LOC device [23,26]. Figure 1 shows the schematic of the fully assembled automatic microfluidic system.

### 2.2. LOC Devices

Two different types of multi-well LOC devices were used in this study. The ibidi LOC has three independent channels, each one containing seven wells with an internal volume of 45 μL per channel. Each well has a bottom diameter of 800 µm and a volume of 3.5 μL. The device has a removable coverslip that allows for individual seeding in each well and facilitates cell recovery at the end of the experiment by removing it. The device is made from a patented bio-inert synthetic polymer designed for single use [27].

**Figure 2:**
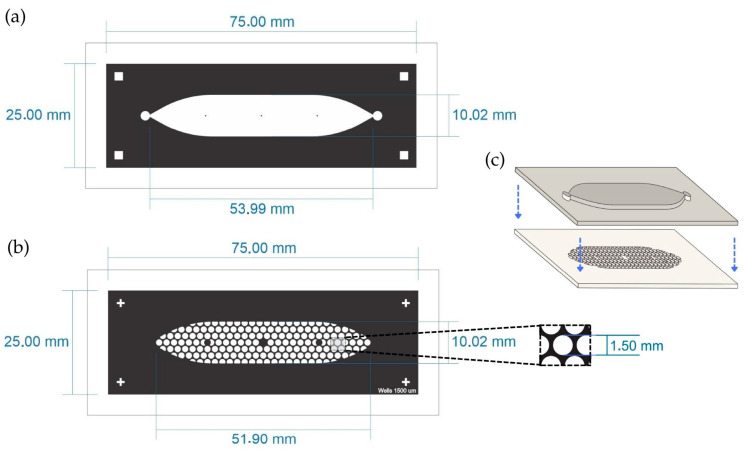
Schematic representation of the PDMS LOC. (**a**) Top layer containing the elongated cistern (53.99 mm × 10.02 mm) with inlet and outlet ports. (**b**) Bottom layer showing the array of circular wells distributed in a hexagonal pattern within the cistern area. The inset shows the diameter of the well (1.5 mm). (**c**) Assembly of both PDMS layers with plasma bonding. The blue arrows indicate the direction of assembly of both layers.

The PDMS LOC consists of two layers. The top layer features an elongated cistern (54 mm in length) with an internal volume of 200 μL, connected to a single inlet and outlet, both fitted with Luer connectors. The bottom layer contains approximately 150 wells, each with a diameter of 1500 µm, distributed in a hexagonal pattern (Figure 2a,b). The design is adaptable and can be modified to present additional channels if required.

The design of the PDMS LOC was optimized to maximize experimental throughput while maintaining conditions suitable for suspension cell culture. The well diameter of 1500 µm and the inclusion of approximately 150 wells per device were adapted from designs in the spheroid formation literature, as these systems similarly rely on gravity to promote the self-assembly of cell clusters [28,29]. To maximize the number of replicates within the device footprint, a hexagonal pattern was chosen for the well array, a well-established strategy for optimizing spatial density in microfluidic designs [30]. This approach, built upon fabrication techniques previously established by our group [31], results in a high-density device capable of supporting numerous, statistically robust replicates for long-term proliferation studies.

The manufacturing process for the PDMS LOC began with a detailed CAD design that was transferred to a photopolymer Flexcel mold (F-mold) from Eastman Kodak. The mold was cured for 72 h at 40 °C using the process described by Bourguignon et al. [12]. A mixture of epoxy resin and curing agent (2:1) was then poured into the F-mold to produce an epoxy resin mold (ER mold). This mixture, commercially known as Cristal-Tack (Novarchem S.A., Buenos Aires, Argentina), was used to replicate the design in high relief following the protocol established by Olmos et al. [32]. Once the ER mold solidified after three days, the F-mold was removed, and an acrylic frame was added.

The PDMS replicas were prepared by mixing the curing agent and base in a 1:10 ratio (Sylgard^®^184, Dow Corning, Midland, MI, USA). The PDMS mixture was vacuum treated for 15 min, poured into the ER mold, and cured at 40 °C for 24 h. After curing, the top and bottom layers were demolded and permanently bonded using plasma-enhanced chemical vapor deposition (PECVD) to ensure a strong seal and structural integrity, the assembly can be seen in Figure 2c. Finally, Luer connectors were inserted into the top layer’s inlet and outlet ports to enable fluid flow into the device.

### 2.3. Cell Line Culture

The suitability of each LOC device was tested using the Jurkat immortalized human T lymphocyte cell line provided by the Lighthouse Core Facility. Jurkat cells were cultured in T-25 flasks with complete Advanced RPMI 1640 medium supplemented with 4% fetal bovine serum (FBS; Life Technologies GmbH, Darmstadt, Germany), 1% penicillin-streptomycin (10,000 units/mL penicillin, 10,000 µg/mL streptomycin), and 1% L-glutamine (Gibco, Waltham, MA, USA). The cells were maintained at 37 °C in a humidified atmosphere with 5% CO_2_ and passaged every three days [33].

### 2.4. Isolation and Activation of Human Donor T Cells

This study was approved by the Ethics Committee of the Albert-Ludwigs-Universität Freiburg. T cells were isolated from buffy coats using a negative isolation strategy [34]. Buffy coats were first collected in 50 mL Falcon tubes, and 29 mL of resuspension buffer (PBS/EDTA) was added for every 6 mL of blood, resulting in a final volume of 35 mL per Falcon tube. The diluted blood cell suspension was carefully layered over 15 mL of Ficoll in separate Falcon tubes. The samples were centrifuged at 400× *g* for 30 min at 18–20 °C using a swinging bucket rotor with the brake function disabled. After centrifugation, the top plasma layer was removed, and the intermediate layer containing peripheral blood mononuclear cells (PBMCs) was carefully collected. The PBMCs were resuspended in 40 mL of resuspension buffer and mixed before centrifugation at 300× *g* for 10 min at 18–20 °C. After removing the supernatant, the cell pellet was resuspended in 50 mL of resuspension buffer and centrifuged again at 200× *g* for 15 min at 20 °C, after which the supernatant was discarded completely. This wash step was repeated once more to ensure the removal of platelets.

Subsequently, 500 μL (approximately 5 × 10^7^ cells) of the PBMCs were resuspended in isolation buffer (Ca^2+^ and Mg^2+^ free PBS supplemented with 0.1% BSA and 2 mM EDTA). T cells were isolated from this cell suspension using the Dynabeads™ Untouched™ Human T Cells Kit (Thermo Fisher, Waltham, MA, USA), as per the manufacturer’s instructions.

Isolated human T cells were activated using the ImmunoCult™ Human CD3/CD28 T Cell Activator (STEMCELL Technologies, Vancouver, BC, Canada) following the manufacturer’s protocol. Briefly, purified T cells were resuspended in Advanced RPMI 1640 medium, supplemented with 4% fetal bovine serum (FBS), 1% penicillin-streptomycin (10,000 units/mL penicillin, 10,000 µg/mL streptomycin), 1% L-glutamine (Gibco) and 20 ng/mL of human recombinant Interleukin-2 (IL-2). The cells were seeded at a density of 1 × 10^6^ cells/mL in a 6-well plate. To initiate activation, the ImmunoCult™ Human CD3/CD28 T Cell Activator was added to the cell suspension at a final concentration of 25 µL/mL. The plate was then incubated at 37 °C in a 5% CO_2_ humidified incubator. This method utilizes soluble tetrameric antibody complexes that co-crosslink the CD3 and CD28 receptors on the T cell surface, providing the primary and co-stimulatory signals necessary for T cell activation and proliferation in a bead-free system. The activation status of the T cells was assessed after 72 h of culture by flow cytometric analysis of activation markers such as CD25 and CD69 [35].

### 2.5. Cell Seeding and Culture in LOC Devices

Prior to seeding, the PDMS LOC was sterilized by autoclaving. Both the device and the previously supplemented Advanced RPMI 1640 medium (Gibco) were incubated overnight at 37 °C. The following day, the device was first filled with medium, followed by the cell suspension. Both were carefully injected directly into the channel using a pipette, ensuring complete coverage of the area while avoiding bubble formation. The device was then incubated at 37 °C for 1 h.

For the ibidi LOC, two seeding protocols were tested. In the first protocol, the coverslip was attached, and then cell-free Advanced RPMI 1640 medium was injected directly into each channel using a micropipette. After a 2 h incubation at 37 °C, the medium was injected again to remove air bubbles from the wells and interconnections. Finally, the cell suspension was injected into the channels, and after a 1 h incubation, the Luer connectors were filled with cell-free medium [27].

In the second protocol, 2 µL of the cell suspension was dispensed into each well using a micropipette, ensuring no bubbles remained at the bottom. The coverslip was then placed, and after a 1 h incubation at 37 °C, cell-free medium was injected to fill each channel and eliminate any remaining bubbles. The Luer connectors were also filled at this stage [27].

Also, an experiment was performed to evaluate the influence of surface coatings on cell behavior. The channels of the ibidi LOC were prepared with two different coatings. One channel was coated with Poly-L-lysine (0.1 mg/mL), the other was coated with Poly-D-lysine (0.1 mg/mL), and the third one was left uncoated as a control. Following the coating procedure, Jurkat cells were seeded using the channel-based protocol, and the devices were cultured for 150 h using the chosen flow protocol.

The cell concentration was determined using an automated cell counter (TC10 Automated Cell Counter, Bio-Rad Laboratories, Inc., Hercules, CA, USA) with Trypan Blue dye (0.40%, 1.5 mL; Bio-Rad Laboratories, Inc.). For cell seeding, a concentration of 5 × 10^5^ cells/mL was used to ensure optimal growth conditions. Once the seeding protocols were completed, the microfluidic system was connected to the LOC device. In all assays, Advanced RPMI 1640 medium [supplemented with 4% fetal bovine serum (FBS), 1% Pen Strep (10,000 Units/mL penicillin and 10,000 µg/mL streptomycin), and 1% L-glutamine (Gibco) was used.

### 2.6. Cell Culture Under Flow Conditions

To assess the impact of different flow profiles on cell growth, experiments were conducted using the ibidi LOC, which enables simultaneous testing of three distinct conditions. Cells were seeded according to channel-based protocol, and the following flow regimens were applied: Static Control—cells were maintained without flow or media exchange; Constant Flow—a continuous laminar flow was applied at a rate of 2 µL/min, which was selected as it is the lowest stable and accurately measurable flow that our system can maintain; and Intermittent Flow—a pulsatile regimen consisting of a 5 min perfusion at 10 µL/min followed by a 2 h and 55 min no-flow interval, repeated every 3 h.

For the rest of the experiments, a flow rate of 10 μL/min was applied to both LOC devices. The flow rate was applied for 20 min (total volume 200 μL) every 2 h for the PDMS LOC. For the ibidi LOC, the flow rate was applied for 5 min in each channel (total volume 50 μL) every 3 h. The different protocols were applied because the PDMS LOC tends to dry faster, as it has a bigger channel volume.

### 2.7. Imaging

Images were acquired using the Lionheart FX automated microscope [36]. Labware definitions were configured in the Gen5 software version 3.03, with adjustments performed individually for each LOC device due to their differing dimensions. To maintain standard culture conditions (37 °C, 5% CO_2_, and 90% humidity) during long-term experiments, a stage-top incubator (ibidi GmbH, Gräfelfing, Germany) was connected to the microscope.

Brightfield images were captured at 4× magnification every 3 h for the ibidi LOC and every 2 h for the PDMS LOC. The imaging schedule was synchronized with the dispensing protocol. Images were analyzed using custom deep-learning image analysis software developed by our team based on the U-Net [37] and YOLOv8 [38] architectures. Additionally, the TWS plugin for Fiji [39] was employed to validate and compare the results.

### 2.8. Image Processing with the TWS Plugin for Fiji

The images captured from ibidi LOC were uploaded to Fiji (Image J 1.54f) [40]. An image stack was generated for each well using all time points. Using the editing tools, the background outside the well was erased.

Using the TWS plugin for Fiji [39], which applies machine learning algorithms to classify an image into different classes, the area covered by cells was distinguished from the background. The classifier was trained with a set of eight images, with the cell-covered regions of interest (ROIs) labeled as one class and the background as another class.

After training, each image stack was analyzed using the same classifier, generating probability maps. The resulting images consisted of three layers: the area outside the well, the background, and the area covered by cells. These layers were separated to isolate the one containing the ROIs, which was then automatically thresholded and converted into a binary mask. The analysis tool was used to measure the area covered by the cells using the “Analyze Particles” option. The results were saved as a comma-separated values (CSV) file.

### 2.9. Image Processing with Deep Learning Methods

Deep Learning is a subset of Machine Learning that utilizes multi-layered artificial neural networks to process and learn from data. These networks are trained to recognize patterns by learning from a labeled dataset, where each data point is associated with a specific label or outcome. Once trained, a network (often referred to as a model) can identify the same patterns in new, unlabeled data. In image analysis, depending on the labels in the training dataset and the type of model used, various tasks can be performed, such as:

- Object Detection: Identify and locate objects within an image, providing bounding boxes for each detected object.
- Semantic Segmentation: Classify each pixel in an image into a predefined category, grouping all pixels that belong to the same class as a single entity.
- Instance Segmentation: Similar to semantic segmentation, with the added capability of differentiating between individual objects.

For the ibidi LOC images, a dataset consisting of the previously generated and manually corrected annotations, originally used for the TWS classifier, was used. A U-Net network was employed, characterized by its distinctive U-shaped structure that has been highly successful in binary Semantic Segmentation tasks, especially within biomedical imaging [37]. The training was performed using the FastAI framework [41], which already includes the implementation of U-Net. The performance of the model was evaluated using the Jaccard Index [42] commonly known as Intersection over Union or IoU. It measures the overlap between predicted and labeled areas, with 1 indicating a perfect match and 0 indicating no overlap between the predicted mask and ground truth mask.

For the PDMS LOC images, the Ultralytics YOLOv8 [38] Instance Segmentation model was evaluated. The YOLOv8 architecture is a real-time detection and segmentation model that prioritizes lightweight computation time and efficient resource use over accuracy. Although real-time inference is not required in this environment, the efficient computation allows it to be used without Graphical Processing Unit (GPU) acceleration. This enables a wider range of potential users to access more advanced analysis methods at the cost of slightly lower segmentation accuracy.

A dataset for the PDMS LOC images was generated following an Iterative Self-Training scheme. Initially, a starting model was trained on a small dataset consisting of seven manually labeled images. This initial model was then used to generate predicted labels for an unlabeled dataset. After manual validation and correction, the predictions were added to the dataset, enabling the training of an improved model. This cycle was repeated iteratively until a robust dataset was generated. To evaluate the performance of the YOLOv8 architecture, the IoU was calculated.

### 2.10. Cell Count Estimation from the Image Analysis and Data Analysis

The primary parameter measured was the cell-covered area (µm^2^ per well) over time (hours). Initially, images were preprocessed to exclude the external area of the well using circular shape detection techniques. Each processed image was then analyzed by the corresponding model, generating a mask of the cell-covered area. Using the pixel-to-micrometer scale of the Lionheart FX microscope and assuming a spherical shape for Jurkat cells, with an average radius of 15 μm and a corresponding surface area of 176.71 μm^2^, the estimated cell number was calculated.

Each well of the PDMS LOC has a top radius of 750 μm and a depth of 248 μm. The cell number was estimated by first calculating the volume corresponding to the total area covered. The areas of the different cell clusters were summed and treated as a single circular colony, centered at the bottom of the well. Since the well is shaped like the lower half of a prolate spheroid (an elongated sphere with two equal shorter axes), geometric equations for this shape were applied to determine the height of the cell cluster and, thus, the total volume occupied by the cells. The height of the space between the top of the colony and the top of the well ($h^{\prime }$) was calculated using the following formula:

(1) $$h^{\prime }=c\sqrt{1-\frac{r^{2}}{R^{2}}}$$

where (*c*) is the total height of the well, (*r*) is the radius of the area of the single colony, and (*R*) is the radius of the top of the well. Using this result, the height of the colony (*h*) from the bottom of the well to the top of the colony was determined as:

(2) $$h=c-h^{\prime }$$

Using the calculated colony height (*h*), the total volume occupied by the cells (*V_cells_*) was determined with the corresponding geometric formula:

(3) $$V_{cells}=\pi r^{2}h-\frac{\pi r^{2}h^{3}}{3c^{2}}$$

Finally, the total number of cells was estimated by dividing this calculated colony volume by the estimated volume of a single cell.

For the ibidi LOC, the cell number estimation was simpler as the well bottom is flat. The number of cells was estimated using the following formula:

(4) $$Number\;of\;cells=\frac{Total\;Cell-Covered\;Area\;({µm}^{2})}{Average\;Single-Cell\;Area\;({µm}^{2})}$$

where the Total Cell-Covered Area is the area generated from the image analysis mask, and the Average Single-Cell Area is the estimated surface area of one cell (e.g., 176.71 µm^2^ for a Jurkat cell).

Cell numbers were graphed and analyzed using GraphPad Software (version 8.3.0; CA, USA). [43]. To evaluate variability, row statistics were applied to calculate the mean and the standard error of the mean (SEM) for each time point, averaging all wells within each channel across replicates. The SEM was computed independently for each dataset at every time point. This approach ensured a robust evaluation of variability and measurement precision, enabling a detailed comparison across time points.

## 3. Results

### 3.1. Deep Learning Methods for Analysis

The U-Net architecture was trained on a dataset containing 629 images from the ibidi LOC, of which 125 were used as a validation set. The IoU was selected as the primary metric for this binary segmentation task. After training, the resulting model achieved an average IoU score of 0.779 on the validation set, reflecting the similarity between the predicted and ground-truth labels.

For the YOLOv8 architecture, a dataset of 231 images from the PDMS LOC was generated using the Iterative Self-Training process, with 31 images designated for validation. The final YOLOv8 model, trained on this dataset, achieved an IoU score of 0.873 on the segmentation task against the validation set.

**Figure 3:**
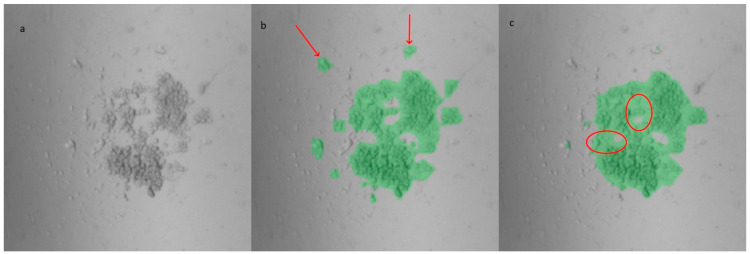
Masks generated from an image of the custom-made PDMS LOC: (**a**) original image, (**b**) YOLOv8m-segm model output, and (**c**) U-Net model output. Compared to YOLOv8, the U-Net masks exhibit smoother edges but encompass more background (highlighted by circles). However, the U-Net model tends to miss certain cells located farther from the center, which are successfully identified by the YOLOv8 masks (indicated by arrows).

The switch in model for the analysis of the PDMS LOC images reflects an exploration of alternative deep learning models that could be more suitable for different environments and expertise levels. An additional U-Net model was trained on the same dataset used for YOLOv8, resulting in an IoU score of 0.912. Despite this higher score, the masks generated by both models showed no significant differences in the analysis of the same images. For comparison, four wells from an experiment conducted using the PDMS LOC, in which 33 images were acquired per well (totaling 132 images), were analyzed. The percentage of the well area covered by the mask was calculated for both models. Although slight visual differences between the masks were observed in some instances, as shown in Figure 3, the average difference in well area coverage between the models was only 0.69%, with a standard deviation of 0.60%. Additional details regarding the training and datasets for both U-Net and YOLOv8 can be found in Supplementary Material S1.1.

**Figure 4:**
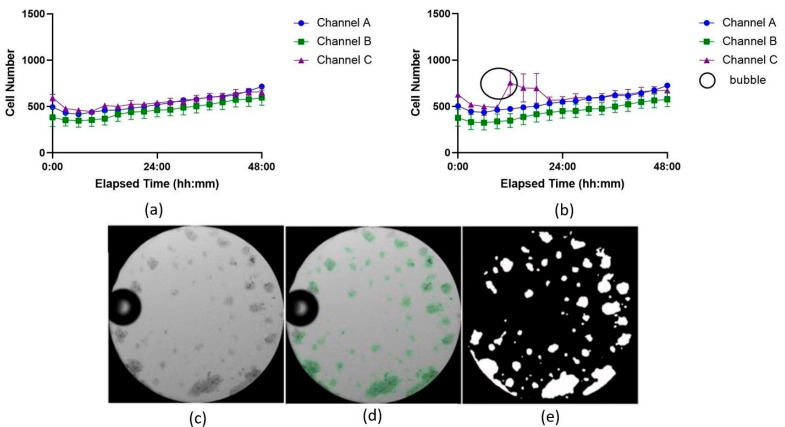
Growth curves showing cell proliferation over time analyzed with two image analysis tools: (**a**) U-Net model and (**b**) Trainable WEKA Segmentation (TWS) plugin for Fiji. The three independent channels of the ibidi LOC were used as technical replicates to culture Jurkat cells under identical conditions and are denoted here as Channel A, B, and C. Each time point represents the average of 7 wells, with the variation shown as the standard error of the mean (SEM). In (**b**), higher SEM variation at most time points suggests less accuracy compared to (**a**). The black circle highlights a bubble’s shadow misidentified as cell area by TWS. (**c**) Microscope image showing the bubble. (**d**) U-Net mask highlighting cells as regions of interest. (**e**) Area detected by the U-Net model, excluding the bubble as cell area.

An experiment using Jurkat cells was conducted in the ibidi LOC to compare image analysis performance between the U-Net model and the TWS. The analysis of the SEM between the two datasets, generated from the same images but analyzed with different methods, revealed differences in the precision of the results (see Supplementary Material S1.3; Table S3). The results obtained with the U-Net model, shown in Figure 4a, displayed lower SEM values compared to those obtained with TWS, shown in Figure 4b.

Although not all time points followed this trend, with some intervals showing similar or slightly higher SEM values for TWS, many of the measurements from the U-Net model consistently exhibited reduced variance. For example, at 21 h in channel C, the SEM for the U-Net model was 16.024, while the SEM for TWS was higher at 33.606. This pattern of reduced variability was observed across most time points, indicating that the U-Net model produced more consistent results, with fewer fluctuations in replicate measurements.

A common challenge in microfluidics, particularly when working with LOC devices that have narrow channels or small areas, is bubble formation. These bubbles are difficult to remove without washing away the suspended cells. When a bubble forms inside the LOC device, it can be misidentified as a cell-covered area, potentially skewing the results. One key advantage of the U-Net model is its ability to more accurately distinguish between bubbles and cell-covered regions, leading to more precise and reliable outcomes, as shown in Figure 4c–e.

Based on the software comparison results, the following experiments performed in the ibidi LOC were analyzed using the U-Net model. All experiments performed with the PDMS LOC were analyzed using the YOLOv8 model.

### 3.2. Comparison of LOC Devices

Despite both LOC devices sharing similar design principles, each one has distinct characteristics as shown in Table 1.

**Figure 5:**
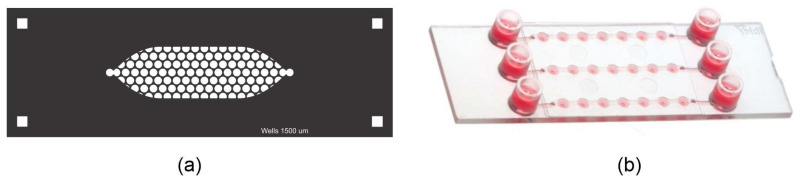
(**a**) Custom-made PDMS LOC and (**b**) μ-Slide Spheroid Perfusion from ibidi ^®^.

For instance, the ibidi LOC has 21 wells distributed across three channels, and as it is commercially manufactured, its dimensions are consistent. On the other hand, for the PDMS LOC, with approximately 150 wells, only 33 wells can be imaged at a time due to the limitations of the microscope, which can only image wells that are properly aligned. Figure 5 illustrates the differences between the two designs.

**Figure 6:**
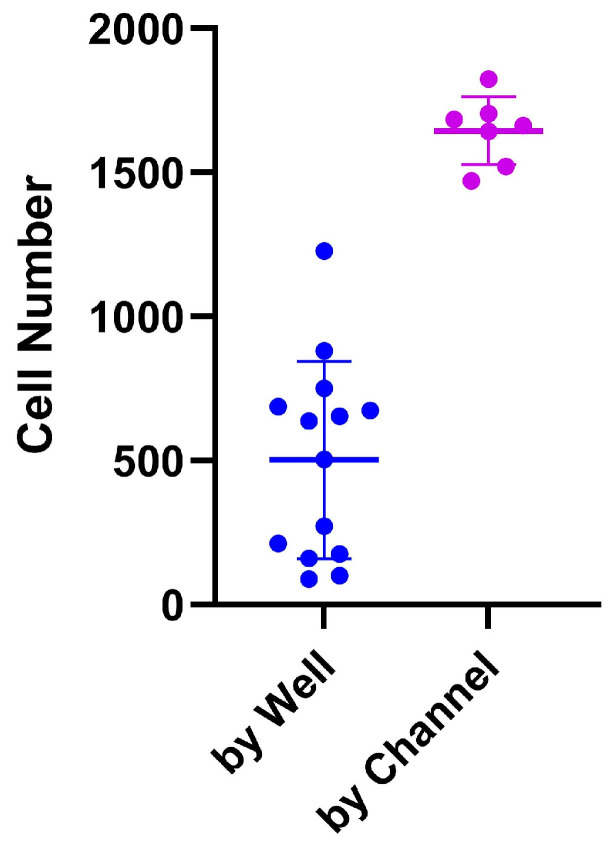
Comparison between two seeding protocols in the ibidi LOC: seeding by well and seeding by channel. Seeding by well shows more variation in cell number compared to seeding by channel, which demonstrates more consistent results. The variation is shown as the standard deviation (SD).

The PDMS LOC permits only a single seeding method due to the absence of a removable coverslip. In this design, cells must be introduced exclusively through the inlet, which leads to a single large reservoir. In contrast, the ibidi LOC includes a detachable coverslip, enabling two distinct seeding approaches: individual well seeding and channel-based seeding. For these experiments, a cell suspension of 5 × 10^5^ cells/mL was prepared, and 45 µL of this suspension (containing a total of 22,500 cells) was introduced into each channel. This volume was distributed across seven individual micro-wells, resulting in an average initial seeding density of approximately 3214 cells per well. As shown in Figure 6, seeding by well led to greater variability in cell number per well, whereas channel-based seeding provided more consistent and accurate distribution, in line with the expected calculations. Additionally, the ibidi LOC simplifies cell recovery, as the removable coverslip allows easy access to each well, facilitating efficient collection of the contents.

Regarding the material, both devices use biocompatible polymers. The PDMS LOC allows reuse after proper sterilization, while the ibidi LOC, which uses a proprietary bioinert synthetic polymer, is designed for one-time use only.

**Figure 7:**
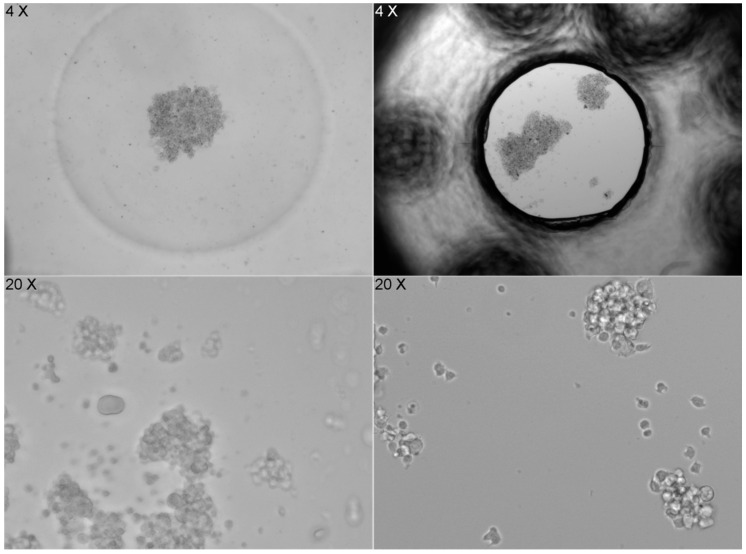
Image series that compares cell distribution in two different LOC devices. On the left, images from the PDMS LOC, which has a concave (U-shaped) bottom, show cells accumulating in the center at 4× magnification, resulting in a more homogeneous distribution. However, at 20× magnification, the cells cannot be properly focused due to the lack of a flat surface. On the right, images from the ibidi LOC, with a flat bottom, show a random and heterogeneous cell distribution at 4× magnification, but images taken at 20× magnification are sharp and clear, allowing for better focus. The contrast highlights the impact of the bottom surface shape on cell distribution and imaging clarity.

The shape of the well bottom also significantly affects how cells settle. The flat bottom of the ibidi LOC leads to random and heterogeneous cell distribution, while the concave (U-shaped) bottom of the PDMS LOC results in a more homogeneous distribution. However, when imaging at 20× magnification, cells in the PDMS LOC cannot be properly focused due to the lack of a flat surface, a limitation that does not affect the ibidi LOC (Figure 7).

### 3.3. Comparison of Flow Conditions

A fundamental aspect of both LOC devices is their design, which enables cells to settle at the bottom of the wells, facilitating media renewal without dislodging the cells. Nutrient and oxygen delivery to the cells occurs via diffusion, and the designs reduce shear forces that typically affect cells under flow conditions [15,27].

The MFS flow sensor used in this microfluidic system operates at flow rates ranging from 2 µL/min to 80 µL/min. An experiment was performed to assess the influence of different flow rates on cell behavior. In the ibidi LOC, even at the maximum flow rate, cells remained securely at the bottom of the well without being dislodged.

When comparing the different flow protocols, cells maintained under static “No Flow” conditions (green line) exhibited minimal proliferation, with cell numbers remaining largely unchanged. This group experienced a significant cell loss event around the 38 h mark, attributed to the chip drying out, and showed no subsequent recovery.

**Figure 8:**
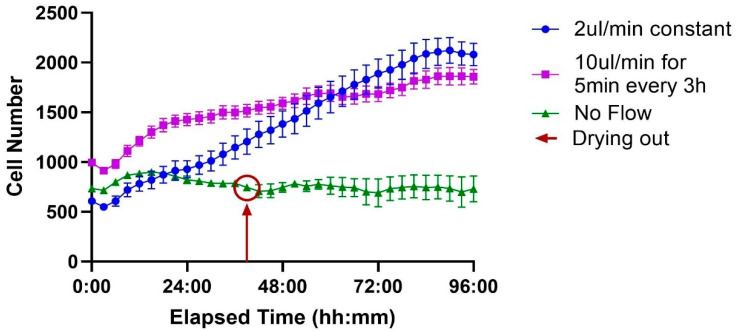
Effect of flow conditions on cell proliferation over time. Three conditions are compared, each performed on a different channel of the ibidi LOC: no flow, constant flow at 2 µL/min, and intermittent flow at 10 µL/min for 5 min every 3 h. Cells under no flow do not proliferate, with the red circle indicating the point when the channel begins to dry out. In contrast, both constant and intermittent flow conditions support robust cell growth, exhibiting similar proliferation rates. Each time point represents the average of 7 wells, with the variation shown as the standard error of the mean (SEM).

In contrast, both perfusion-based conditions supported sustained and significant cell growth. Cells under constant flow (2 µL/min, blue line) showed steady proliferation throughout the experiment. Similarly, the intermittent flow regimen (10 µL/min for 5 min every 3 h, purple line) also promoted robust growth. These results demonstrate that exchange is critical for supporting sustained cell proliferation compared to a static environment (Figure 8).

The effect of different flow conditions on cell proliferation over 96 h was analyzed using a mixed-effects model (REML), with flow rate and time as fixed effects. The analysis revealed a significant Time × Flow rate interaction (F (88, 792) = 28.06, *p* < 0.0001), indicating that the effect of the treatment on cell number depended on the time point. The main effects of Time (F (44, 792) = 57.88, *p* < 0.0001) and Flow Rate (F (2, 18) = 36.88, *p* < 0.0001) were also significant.

Post hoc analysis using Tukey’s multiple comparisons test was performed to compare treatments at each time point (full results are available in Supplementary Material S1.3; Table S4). The results for the final 96 h endpoint are presented here. Both the constant flow (2 µL/min) and intermittent flow (10 µL/min) conditions resulted in significantly higher cell numbers compared to the no-flow control (*p* = 0.0001 and *p* = 0.0004, respectively). However, there was no statistically significant difference between the constant and intermittent flow strategies at this final time point (*p* = 0.7745).

**Figure 9:**
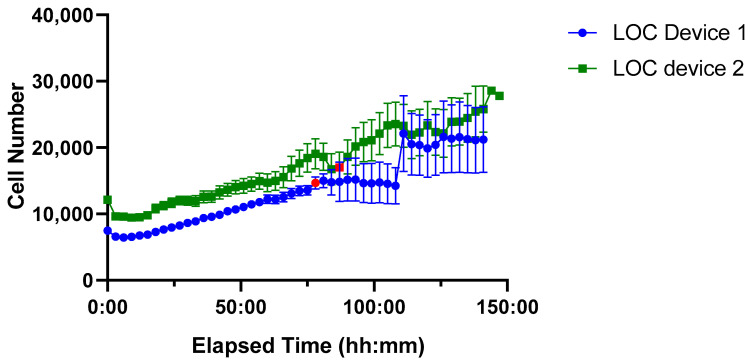
Time-course of Jurkat cell proliferation in two PDMS LOC over ~150 h. Device 1 (blue line, circular markers) and Device 2 (green line, square markers) show sustained cell growth with low variability until the onset of drying, indicated by red markers. Each data point represents the mean cell number per device, with error bars denoting the standard error of the mean (SEM).

Unlike the ibidi LOC, the PDMS LOC features a single-channel design, making it unnecessary to test multiple flow rates; instead, the intermittent flow protocol (10 µL/min for 5 min every 3 h was applied as it provided robust growth with minimal medium consumption in the ibidi LOC. A time-course analysis of cell proliferation was performed over approximately 150 h using two independent devices (Figure 9).

Both PDMS LOCs supported higher initial seeding densities (~10,000 cells) compared to the ibidi LOC, reflecting the greater volume capacity of the custom-made design. Sustained cell growth was observed in both cases; however, increased variability emerged at later time points, particularly after the red marker indicating the onset of device drying, a factor that likely compromised culture stability during extended incubation.

### 3.4. Proliferation of Cells in the LOC Devices

An experiment was performed to test if Poly-L-lysine and Poly-D-lysine coatings could improve image stability without compromising cell growth in the ibidi LOC. While these are applied to facilitate cell adhesion, they can negatively impact cell proliferation, a known side effect reported in the literature [44].

**Figure 10:**
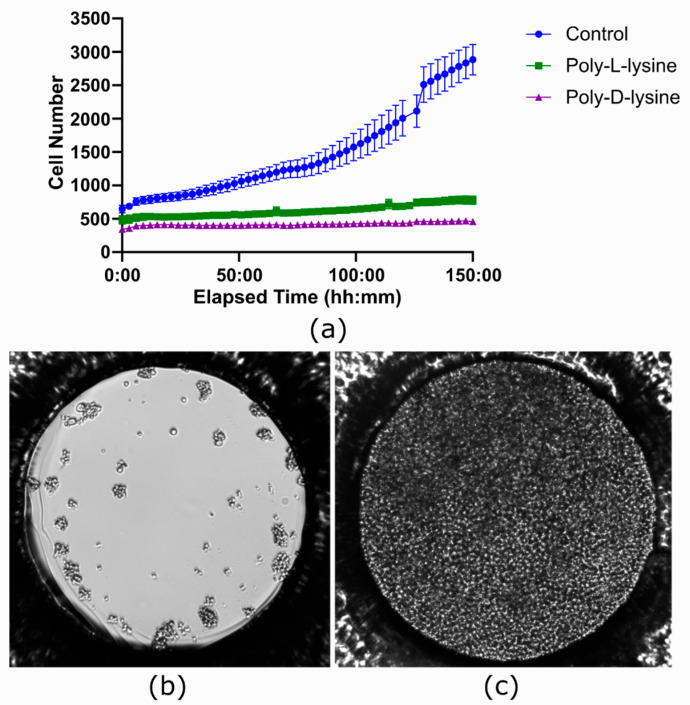
Effect of coating with polylysine in the ibidi LOC. (**a**) Growth curves comparing three conditions, each performed in a different channel of the ibidi LOC: Poly-L-lysine coating, Poly-D-lysine coating, and no coating as a control. In the coated channels, no cell proliferation is observed, whereas the uncoated channel shows significant proliferation of Jurkat cells. Each time point represents the average of 7 wells, with the variation shown as the standard error of the mean (SEM). (**b**) Image taken at the beginning of the experiment showing the initial state of the cells. (**c**) Image taken at the end of the experiment, where the entire surface area of the uncoated well is covered with cells.

As seen in Figure 10a, no cell proliferation was observed in the coated channels. In contrast, by the end of the experiment, the channel that was not coated showed complete coverage of the well surface by cells (Figure 10c), with an average total count of 2883.04 cells across seven wells. Cell viability was assessed using the trypan blue protocol and the Bio-Rad TC10™ automated cell counter. The initial viability was 70%, but after 150 h it changed to 14% for Poly-D-lysine treatment, 56% for Poly-L-lysine treatment, and 92% for the untreated condition.

### 3.5. Proliferation of Human T Cells from Healthy Donors

**Figure 11:**
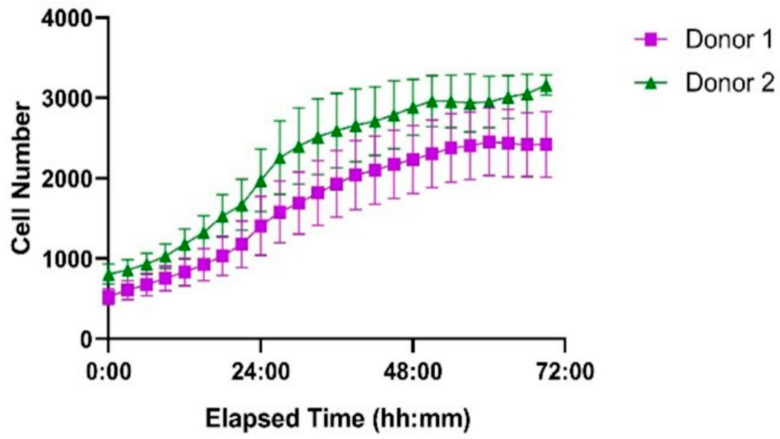
Proliferation of primary human T cells from two healthy donors in the ibidi LOC over 72 h. Cells were seeded at identical initial densities. Donor 1 (purple squares) and Donor 2 (green triangles) both showed successful proliferation, with Donor 2 exhibiting a higher growth rate. Each point represents the mean cell number per time point, and error bars indicate the standard error of the mean (SEM) based on measurements from seven wells.

The ibidi LOC system was further evaluated using primary human T cells isolated from two healthy donors, seeded at the same initial density as Jurkat cells. Based on the results from the Jurkat cell experiments, uncoated channels were used for this study to ensure optimal proliferation conditions. As shown in Figure 11, both donor-derived T cell populations successfully proliferated within the microfluidic environment. Donor 2 exhibited a more pronounced proliferative response compared to Donor 1, particularly between 24 and 48 h. These results demonstrate that the system supports the expansion of primary human T cells with donor-dependent variability.

## 4. Discussion

The microfluidic system used in this research utilizes pressure-driven flow control technology, managed by the OB1 pressure controller in combination with the MFS flow sensor for feedback. The ESI software version 3.07.03 enabled precise tuning of Proportional (Fast/Stable) and Integral (Sensitive/Smooth) parameters, which, using a standardized algorithm, adjusted the pressure based on the sensor’s readings [23,24,25].

Compared to other systems, such as syringe or peristaltic pumps, the microfluidic system presented here offers several advantages. Notably, it can deliver small volumes of liquid, with a minimum flow rate of 2 μL/min, while maintaining precise, pulse-free flow control and a response time of up to 35 ms. This level of precision and consistency is critical for ensuring accurate experimental conditions, particularly in biological assays where flow disturbances can impact cell behavior or disrupt microenvironmental stability. Pulse-free flow is especially advantageous, as it prevents fluctuations that could introduce shear stress or uneven distribution of nutrients and reagents, thereby improving the reliability and reproducibility of experimental outcomes [1,45,46].

Furthermore, incorporating MUX valves into the setup enables versatile functionality, including reagent injection, programmed perfusion, and sequential delivery in microfluidic experiments. The system can inject up to twelve different liquid samples from separate reservoirs into a single microfluidic line or deliver one liquid sample to twelve different microfluidic lines, all within less than 156 ms [18,26].

In this study, we also compared deep learning models with the TWS plugin for Fiji, finding that the models yielded more consistent cell detection results across experiments. This consistency can be attributed to the model’s ability to recognize image patterns and better distinguish cells from artifacts, whereas TWS often struggled, especially with bubbles in the imaging field, leading to less precise results. Deep learning techniques could become the preferred method for image analysis in experiments where precision and consistency in cell quantification are crucial, as they reduce user-dependent variability and improve reproducibility [47].

A key practical consideration in image analysis is the demand for computational resources. While models like U-Net require powerful GPUs, our work shows that the modern YOLOv8 architecture achieves comparable performance with greater efficiency. This makes advanced, local image analysis feasible without specialized hardware. This finding suggests that high-quality, deep learning-based analysis is becoming more accessible. It parallels the success of tools like TWS, which became widely used due to its accessibility on standard CPUs. However, deep learning models offer a crucial advantage: superior performance. While TWS provided a gateway to machine learning, architectures like YOLOv8 deliver a significant leap in accuracy over these traditional methods. Therefore, by lowering the hardware requirements, efficient models like YOLOv8 become more accessible to researchers without an IT background.

In addition to performance, deep learning models offer distinct advantages in automation and efficiency. Once trained, a deep learning model fully automates the segmentation of new images, removing the need for the manual correction steps often required for refining TWS results on a per-experiment basis. This automation not only reduces analysis time but also improves reproducibility by eliminating user-dependent variability. Furthermore, advanced deep learning strategies, such as iterative self-training, can significantly reduce the initial manual annotation effort required to build a robust model.

Regarding usability, TWS currently offers a more integrated training process. However, this usability gap is closing as new tools emerge to streamline the workflow for models like YOLO. A future Fiji plugin with a pre-trained YOLO model, for example, would further increase accessibility. A limitation of the models in this study is their device-specific training; application to different optical systems would require retraining to ensure optimal performance.

While TWS currently offers a more user-friendly training process, including labeling, dataset generation, and model training, new tools are continuously emerging that aim to streamline these steps for YOLO and similar methods, bridging the existing usability gap. In the future, a dedicated Fiji plugin with a pre-trained YOLO model, for example, would further increase accessibility. It is important to note that because the models developed in this study were trained on device-specific images, their application to different optical setups would likely require retraining to ensure optimal performance.

In following studies, deep learning-based analysis can be expanded to include additional visual metrics, such as cell roundness and brightness, which are crucial for detecting subtle changes in cell morphology. These metrics will be especially valuable in experiments conducted at higher magnifications, as they provide critical insights into cell health, activation, and viability.

LOC devices offer significant advantages in cell culture by utilizing microchambers that allow cells to settle at the bottom of the wells. The design ensures that the media can be renewed without the risk of dislodging or flushing out the cells, which is a common challenge in traditional suspension culture methods. Nutrient and oxygen delivery to the cells is facilitated by diffusion, which effectively sustains cell viability and proliferation within the microenvironment of the LOC device, as supported by previous studies [9,14,15,16].

Another key benefit of the LOC devices is their compact size, which translates into reduced reagent consumption compared to conventional cell culture methods. For instance, in a standard 96-well plate, each well requires 200 µL of medium, resulting in a total media usage of at least 19.2 mL per plate. In contrast, the PDMS LOC significantly reduces this volume, requiring only 200 µL of medium per device, while the ibidi LOC further minimizes consumption to 45 µL per channel, with a total of 135 µL per device [10]. This considerable reduction in medium volume not only lowers experimental costs but also allows for more efficient use of reagents, which is particularly advantageous in long-term experiments or studies involving expensive or scarce materials.

This research, which utilizes two types of LOC devices, underscores each device’s distinct advantages concerning specific experimental objectives. The PDMS LOC enables multiple repetitions of the same experiment, enhancing reproducibility, though it can only test a single condition at a time. It also allows a higher rate of cell production, accelerating biological research and providing a larger pool of cells for experiments or therapeutic applications. Increased cell proliferation offers potential benefits in microbial bioprocesses or the production of antibodies, reducing costs and enhancing scalability [10,11,13]. Furthermore, the PDMS LOC’s in-house production facilitates easy modifications. This can increase the number of channels and establish a standardized design, ultimately simplifying microscope configuration and improving imaging accuracy. However, the lack of a removable coverslip restricts seeding flexibility and straightforward cell recovery, making it challenging to retrieve samples entirely.

Many LOC devices are constructed from PDMS due to its excellent biocompatibility, transparency for microscopy applications, and suitability for convenient prototyping. While other biomaterials such as polystyrene and polylactic acid are also available, PDMS remains one of the most widely used materials because of its flexibility and durability [48]. However, it is important to note that PDMS can absorb small molecules, which may bias experimental results [40]. Therefore, researchers should be aware of the limitations associated with PDMS technology when conducting biological studies. To mitigate this and other potential contamination issues, one effective strategy is to use a new device for each experiment. This single-use approach was adopted in our study; although PDMS is an autoclavable material suitable for reuse, each PDMS LOC was used only once to ensure experimental conditions were directly comparable to the single-use ibidi LOC. Additionally, if a PDMS LOC is reused, it is advisable to compare the results of the initial experiment with subsequent ones to assess any discrepancies [12,49].

In the case of the ibidi LOC, its design enables the simultaneous testing of three conditions, enhancing its versatility for comparative analyses. For example, various cytokine assays can be conducted by applying different cytokine cocktails or by assessing cytokine production after the experiment through medium recovery. Additionally, cytotoxicity assays and T-cell activation studies can be performed by testing different concentrations or doses of drugs or activators, as well as varying the timing of application, which is facilitated by the automated microfluidic system. Future studies will expand to evaluate cell death and activation as functional readouts of cells cultured within these devices. This approach highlights the potential of this microfluidic system to advance immunotherapy research, enabled by the device’s flat surface, which facilitates imaging at 20× magnification.

In addition, the ibidi LOC offers greater versatility with its removable coverslip, supporting two seeding methods: individual well seeding and channel-based seeding. This design facilitates easier access to and collection of content from each well, streamlining downstream analyses.

Flow rate is a critical parameter for suspension-based cultures like T-cells in microfluidic systems. The constant flow rate of 2 µL/min was selected as it represents the lowest stable and accurately measurable rate for the MFS flow sensor used in our setup. We tested this continuous perfusion to determine if a constant supply of fresh medium, despite its higher consumption, would significantly enhance T-cell proliferation compared to an intermittent supply. Our results demonstrate that while both perfusion strategies led to significantly superior cell expansion compared to the static no-flow control, there was no statistically significant difference in the final cell number between constant and intermittent methods. This suggests that both approaches effectively enhance nutrient and waste exchange. Constant flow likely provides a stable supply, while intermittent pulses may function by periodically flushing the system to “reset” the local environment. Critically, the intermittent regimen consumes over seven times less medium. Given this comparable efficacy, intermittent flow emerges as the more advantageous and resource-efficient strategy for robust T-cell expansion [50,51].

In our microfluidic system, coating with Poly-D(L)-Lysine improved imaging stability but negatively affected cell proliferation, indicating that the drawbacks outweighed the intended benefits. As a synthetic positively charged polymer, polylysine is known for its strong cell-capturing properties, making it a popular choice for promoting adhesion in suspension cell experiments. By coating surfaces with this polymer, interactions between positively charged molecules and negatively charged cell membranes are facilitated, thus enhancing cell attachment. This is particularly beneficial for imaging and analyzing cells in microscopy and long-term studies, as it improves visibility and stability [44].

However, recent studies indicate that while polylysine is considered an inert compound, it can unintentionally alter the activity of membrane proteins, such as the T-cell antigen receptor (TCR), and hinder normal cellular processes. Specifically, its tendency to anchor cells to the bottom of culture channels may inhibit the formation of rosette-like structures, thereby impairing the cells’ ability to receive signals from neighboring cells, which could result in slowed proliferation [44].

Regarding viability, studies suggest that Poly-L-Lysine can induce distinct mitochondrial-dependent apoptosis in various human cell types [49]. Additionally, the cytotoxic effects of this polymer have been found to correlate with both treatment duration and concentration, which could have significantly impacted the cells, given the long-term nature of our experimental setup [52]. Moreover, as Jurkat cells exist in suspension, adhesion to surfaces can deform the cells and alter their normal function, potentially leading to cell death [53].

This suggests that while polylysine can enhance cell adhesion and prevent washout in flow-based or perfusion experiments, it may also impede growth under certain conditions. In our system, image stability was not a limiting factor since the entire well could be captured, and due to the design of the LOC devices, cell washout did not occur. Given that proliferation was inhibited, the use of polylysine was therefore discarded. This highlights the need for careful consideration of the effects of polylysine in experimental designs, particularly when the goal is to foster robust cellular growth and proliferation.

The microfluidic system presented in this study demonstrates its versatility by successfully supporting both cell lines and primary cells. This capability is particularly advantageous in immunotherapy research, where the use of primary cells is crucial for more accurate models of human immune responses. Future studies will expand the potential applications of the presented LOC devices by evaluating cell-killing efficiency as a functional readout after culturing cells in these devices. This will provide valuable insights into how the devices perform under conditions where cytotoxic assays are required, such as in drug screening or immunotherapy studies. Such functional evaluations are critical for determining the translational relevance of the devices to real-world therapeutic applications.

## 5. Conclusions

This study highlights the significant advancements offered by an automated microfluidic system that employs pressure-driven flow control technology, enhancing precision and consistency in biological assays. The integration of a pressure controller and flow sensor, combined with optimized control parameters, ensures stable flow conditions crucial for maintaining cell behavior and microenvironmental stability. The system’s ability to deliver small volumes with pulse-free flow reduces shear stress and variability in nutrient distribution, thereby improving the reliability and reproducibility of experimental outcomes. Furthermore, the use of deep learning-based software for image analysis minimizes variability in results, reduces analysis time, and lowers the incidence of false positives. These advantages suggest that deep learning tools may become the preferred choice for image analysis in experiments requiring high precision and consistency in cell quantification.

A key feature of this system is the use of LOC devices specifically designed to allow suspension cells to settle at the bottom of the wells. This design enables media renewal without flushing out the cells, making it particularly advantageous for conducting long-term experiments with suspension cells. These devices provide unique strengths in cell culture applications, ensuring stable conditions and preserving cell viability over extended periods.

Regarding the LOC devices presented in this study, the PDMS LOC facilitates multiple experimental repetitions, enhancing reproducibility. Under optimized conditions, it also supports greater cell proliferation compared to the ibidi LOC. On the other hand, the ibidi LOC offers greater versatility by enabling the simultaneous testing of three different conditions and allowing for cell recovery, which is essential for downstream analyses. Overall, these findings underscore the importance of selecting the appropriate LOC device to align with specific research objectives, paving the way for more effective experimental designs in cellular biology.

## Figures and Tables

**Table 1:** Comparison between LOC devices, custom-made (PDMS) vs. commercial (ibidi ^®^).

Characteristic	Custom-Made PDMS LOC Device	ibidi ^®^ LOC Device
Microscope configuration	Variable (Personalized for each chip according to needs)	Fixed
Wells	150	21
Well diameter	1500 µm	800 µm
Inlets	1 (single channel *)	3 (separate channels)
Internal volume per channel	200 µl	45 µl
Coverslip	Non-removable/fixed	Separate
Recovery of cells	Difficult (Pressure-dependent)	Easy (Coverslip can be removed)
Well bottom	U bottom	Flat bottom
Distribution of cells per well	Homogeneous (Accumulation in the center)	Heterogeneous
Reusable	Yes ^†^	No

* The design is adaptable and can be modified to include additional channels. ^†^ PDMS is an autoclavable material suitable for reuse; however, for this comparative study, each device was used only once to ensure experimental conditions were directly comparable to the ibidi LOC.

## Data Availability

The data presented in this study are available on request from the corresponding author due to confidentiality agreements with the industrial partner.

## Supplementary Materials

The following supporting information can be downloaded at: https://www.mdpi.com/article/10.3390/bios15100693/s1, S1.1: Specifications of the Deep Learning Models; S1.1.1: U-Net for the ibidi® LOC Analysis; Table S1: Specifications of the U-Net for the ibidi ® LOC analysis; S1.1.2: YOLOv8 for the PDMS LOC Analysis; Table S2: Specifications of the YOLOv8 for the PDMS LOC analysis; S1.2: Hardware; S1.3: Results of statistical analysis; Table S3: Statistical Comparison of U-Net vs. TWS Cell Counting. This table shows the mean and standard error of the mean (SEM) for cell counts obtained via image analysis using the two models; Table S4: Tukey’s multiple comparisons of flow rate conditions across experimental rows.

## Abbreviations

The following abbreviations are used in this manuscript:

LOC	Lab-on-Chip
TWS	Trainable Weka Segmentation for Fiji
ACT	Adoptive cell therapy
PDMS	Polydimethylsiloxane
PBMCs	Peripheral blood mononuclear cells
SEM	Standard error of the mean
PBS	Phosphate-buffered saline
Pen Strep	Penicillin streptomycin
RPMI	Roswell Park Memorial Institute Medium
EDTA	Ethylenediaminetetraacetic acid
IL-2	Interleukin-2
