# Supplementary material for: Automated T-Cell Proliferation in Lab-on-Chip Devices Integrating Microfluidics and Deep Learning-Based Image Analysis for Long-Term Experiments
Source: Biosensors (Basel). 2025 Oct 13;15(10):693. doi: 10.3390/bios15100693 (PMC12563272; doi:10.3390/bios15100693)
Supplement: Supplementary file 1 [file biosensors-15-00693-s001.zip › biosensors-3842488-supplementary.pdf]

biosensors

MDPI

Supplementary Material

Automated T-Cell Proliferation in Lab-on-Chip Devices Integrating Microfluidics and Deep Learning-Based Image Analysis for Long-Term Experiments

María Fernanda Cadena Vizuete [1,2,3,4], Martin Condor [5], Dennis Raith [5,6], Avani Sapre [1,5], Marie Follo [7,8], Gina Layedra [9], Roland Mertelsmann [1], Maximiliano Perez [9,10,11]* and Betiana Lerner [9,10,11,*]

1 Mertelsmann Foundation, Freiburg, 79104, Germany; mc294@email.uni-freiburg.de (M.F.C.V); avani@labmaite.com (A.S.); roland.mertelsmann@uniklinik-freiburg.de (R.M.)
2 Facultad de Farmacia y Bioquímica, Universidad de Buenos Aires, Ciudad Autónoma de Buenos Aires,1113, Argentina
3 Faculty of Biology, Albert-Ludwigs-University of Freiburg, Freiburg,79104, Germany
4 CONICET, Instituto de Investigaciones en Microbiología y Parasitología Médica (IMPaM), Universidad de Buenos Aires, Ciudad Autónoma de Buenos Aires, 1121, Argentina
5 LABMaiTE GmbH, Freiburg, 79110, Germany; martin@labmaite.com (M.C.); dennis@labmaite.com (D.R.)
6 Neurorobotics Lab, Department of Computer Science, Albert-Ludwigs-University of Freiburg, Freiburg, Germany
7 Department of Medicine I,, Faculty of Medicine, Medical Center - University of Freiburg, Freiburg, 79106, Germany; marie.follo@uniklinik-freiburg.de (M.F.)
8 Lighthouse Core Facility, Faculty of Medicine, Medical Center—University of Freiburg, Freiburg, 79106, Germany
9 IREN Center, National Technological University, Buenos Aires 1706, Argentina; gina-layedra@hotmail.com (G.L.)
10 Collaborative Research Institute Intelligent Oncology (CRIION), Freiburg, 79110, Germany
11 Department of Electrical and Computer Engineering, Florida International University, Miami, FL 33174 USA
* Correspondence: maxperez@fiu.edu(M.P.); belerner@fiu.edu(B.L.)

Received: 12 August 2025
Revised: 13 September 2025
Accepted: 2 October 2025
Published: 13 October 2025

Citation: Cadena Vizuete, M.F.; Condor, M.; Raith, D.; Sapre, A.; Follo, M.; Layedra, G.; Mertelsmann, R.; Perez, M.; Lerner, B. Automated T-Cell Proliferation in Lab-on-Chip Devices Integrating Microfluidics and Deep Learning-Based Image Analysis for Long-Term Experiments. *Biosensors* **2025**, *15*, 693. https://doi.org/10.3390/bios15100693

Copyright: © 2025 by the authors. Licensee MDPI, Basel, Switzerland. This article is an open access article distributed under the terms and conditions of the Creative Commons Attribution (CC BY) license (https://creativecommons.org/licenses/by/4.0/).

S1. Supplementary Data

S1.1. Specifications of the Deep Learning Models

S1.1.1. U-Net for the ibidi® LOC Analysis

Table S1. Specifications of the U-Net for the ibidi ®LOC analysis.

Concept	Value
Framework	FastAI (v2.7.13) U-Net
Task	Binary segmentation of cells and background
Training dataset	629 microscopy images. 20% used as a validation set
Preprocessing	Images were cropped to fit the well.<br>Dimensions: 539x544px
Hyperparameters	Backbone: Resnet34<br>Optimizer: Adam<br>Loss function: Cross Entropy<br>Batch size: 4<br>Learning rate: 0.001<br>Epochs: 16
Augmentation	Random horizontal flips. Vertical flips. Rotations (max 180°) scaling, and crops.
Accuracy	IoU score of 0.7785 on the validation set

S1.1.2. YOLOv8 for the PDMS LOC Analysis

Parameters not listed here were set with the default values assigned by Ultralytics.

Table S2. Specifications of the YOLOv8 for the PDMS LOC analysis.

Concept	Value
Framework	Ultralytics (v8.2.70) YOLOv8m-segm
Task	Instance segmentation of cell clusters
Training dataset	231 microscopy images. 15% used as a validation set
Preprocessing	Images were downscaled from the original size.<br>Dimensions: 640x640px
Hyperparameters	Optimizer: AdamW<br>Loss function: Cross Entropy<br>Batch size: 16<br>Learning rate: 0.01<br>Epochs: 200
Accuracy	IoU score of 0.8729

S1.2. Hardware

The models were trained on a machine equipped with an AMD Ryzen 9 5950X 16-core processor with 124 GB RAM and an NVIDIA GeForce RTX 3090 GPU with 24GB of memory.

biosensors

MDPI

S1.3. Results of statistical analysis

Table S3. *Statistical Comparison of U-Net vs. TWS Cell Counting. This table shows the mean and standard error of the mean (SEM) for cell counts obtained via image analysis using the two models.*

Elapsed Time (hh:mm)	U-Net model									WEKA segmentation plugin								
	Channel A			Channel B			Channel C			Channel A			Channel B			Channel C		
	**Mean**	**SEM**	**N**	**Mean**	**SEM**	**N**	**Mean**	**SEM**	**N**	**Mean**	**SEM**	**N**	**Mean**	**SEM**	**N**	**Mean**	**SEM**	**N**
**00:00**	494.952	16.040	4	383.464	98.763	3	595.911	36.715	3	504.220	14.214	4	377.212	90.125	3	626.735	28.556	3
**03:00**	432.403	10.925	4	353.191	62.119	3	478.202	23.600	3	445.569	10.289	4	332.390	80.343	3	519.812	17.365	3
**06:00**	415.054	7.898	4	347.116	68.867	3	460.198	27.158	3	435.173	3.264	4	324.696	79.632	3	498.256	14.676	3
**09:00**	439.683	12.777	4	355.866	69.903	3	447.292	24.387	3	461.820	12.711	4	338.022	78.838	3	491.250	9.319	3
**12:00**	457.644	16.153	4	369.895	70.123	3	510.619	20.914	3	473.430	12.536	4	347.334	75.759	3	756.082	132.081	3
**15:00**	463.131	10.489	4	415.925	75.199	3	501.256	12.128	3	490.248	9.640	4	386.803	82.257	3	699.144	151.647	3
**18:00**	481.721	10.495	4	441.001	80.085	3	524.276	41.612	3	503.547	6.743	4	415.170	84.047	3	695.395	159.694	3
**21:00**	499.961	16.630	4	446.099	74.717	3	525.827	16.024	3	531.591	13.979	4	435.213	86.858	3	568.208	33.606	3
**24:00**	520.019	15.111	4	462.494	72.498	3	540.342	23.131	3	550.310	13.036	4	449.685	76.915	3	569.358	36.613	3
**27:00**	540.788	12.990	4	465.267	63.927	3	552.939	36.746	3	558.724	7.575	4	451.781	71.936	3	595.409	41.929	3
**30:00**	569.436	24.034	4	489.607	79.057	3	553.994	33.665	3	588.085	17.904	4	472.483	66.601	3	590.665	25.773	3

**33:00**	578.390	12.637	4	502.753	73.236	3	581.077	41.646	3	595.976	8.699	4	476.111	65.400	3	599.012	40.286	3
**36:00**	601.680	7.339	4	523.353	59.492	3	601.668	40.042	3	620.348	11.793	4	498.285	64.165	3	633.678	38.213	3
**39:00**	607.307	14.886	4	546.555	79.016	3	612.038	31.905	3	615.448	15.042	4	523.673	75.684	3	631.148	52.556	3
**42:00**	625.816	11.202	4	573.181	77.269	3	644.135	40.470	3	644.666	9.835	4	548.276	69.469	3	655.156	50.788	3
**45:00**	669.002	24.971	4	577.554	76.350	3	657.099	37.478	3	675.276	17.412	4	564.424	79.037	3	673.181	39.599	3
**48:00**	715.239	17.842	4	595.381	81.936	3	656.849	51.026	3	726.201	20.096	4	575.063	78.322	3	671.280	65.567	3

Note: *N represents the number of replicates (wells) used for the analysis. The mean and SEM were calculated for each time point across all three channels of the ibidi® LOC.*

Table S4. Tukey's multiple comparisons of flow rate conditions across experimental rows.

Tukey's multiple comparisons test	Mean Diff.	95.00% CI	Sig.	Summary	Adj. P Value
**Row 1**					
2ul/min constant vs. 10ul/min for 5min every 3h	-387.3	-502.9 to -271.7	Yes	****	<0.0001
2ul/min constant vs. No Flow	-126.5	-245.9 to -7.117	Yes	*	0.0380
10ul/min for 5min every 3h vs. No Flow	260.8	169.3 to 352.3	Yes	****	<0.0001
Row 2					
2ul/min constant vs. 10ul/min for 5min every 3h	-363.2	-484.4 to -242.1	Yes	****	<0.0001
2ul/min constant vs. No Flow	-167.1	-288.7 to -45.51	Yes	**	0.0090
10ul/min for 5min every 3h vs. No Flow	196.1	100.6 to 291.6	Yes	***	0.0004
Row 3					
2ul/min constant vs. 10ul/min for 5min every 3h	-372.7	-552.6 to -192.7	Yes	***	0.0004
2ul/min constant vs. No Flow	-191.9	-356.4 to -27.28	Yes	*	0.0238
10ul/min for 5min every 3h vs. No Flow	180.8	36.63 to 325.0	Yes	*	0.0155
Row 4					
2ul/min constant vs. 10ul/min for 5min every 3h	-390.2	-632.3 to -148.2	Yes	**	0.0029
2ul/min constant vs. No Flow	-147.8	-363.6 to 67.88	No	ns	0.1836
10ul/min for 5min every 3h vs. No Flow	242.4	64.20 to 420.6	Yes	*	0.0109
Row 5					
2ul/min constant vs. 10ul/min for 5min every 3h	-426.0	-672.5 to -179.5	Yes	**	0.0020
2ul/min constant vs. No Flow	-101.7	-335.3 to 131.9	No	ns	0.4542
10ul/min for 5min every 3h vs. No Flow	324.3	170.6 to 478.0	Yes	***	0.0006
Row 6					
2ul/min constant vs. 10ul/min for 5min every 3h	-480.7	-774.3 to -187.2	Yes	**	0.0028
2ul/min constant vs. No Flow	-77.77	-350.5 to 195.0	No	ns	0.6871
10ul/min for 5min every 3h vs. No Flow	403.0	214.0 to 592.0	Yes	***	0.0009
Row 7					
2ul/min constant vs. 10ul/min for 5min every 3h	-498.0	-784.7 to -211.2	Yes	**	0.0017
2ul/min constant vs. No Flow	-16.27	-273.0 to 240.5	No	ns	0.9812
10ul/min for 5min every 3h vs. No Flow	481.7	277.4 to 686.0	Yes	***	0.0004
Row 8					
2ul/min constant vs. 10ul/min for 5min every 3h	-498.3	-816.3 to -180.3	Yes	**	0.0040
2ul/min constant vs. No Flow	56.02	-246.1 to 358.2	No	ns	0.8547
10ul/min for 5min every 3h vs. No Flow	554.3	358.8 to 749.9	Yes	****	<0.0001
Row 9					
2ul/min constant vs. 10ul/min for 5min every 3h	-498.2	-786.5 to -209.9	Yes	**	0.0019
2ul/min constant vs. No Flow	106.8	-162.2 to 375.8	No	ns	0.5223
10ul/min for 5min every 3h vs. No Flow	605.0	408.0 to 802.0	Yes	****	<0.0001
Row 10					
2ul/min constant vs. 10ul/min for 5min every 3h	-469.8	-782.3 to -157.4	Yes	**	0.0050

2ul/min constant vs. No Flow	162.6	-127.0 to 452.3	No	ns	0.2910
10ul/min for 5min every 3h vs. No Flow	632.5	425.3 to 839.6	Yes	****	<0.0001
Row 11					
2ul/min constant vs. 10ul/min for 5min every 3h	-446.1	-774.7 to -117.5	Yes	**	0.0099
2ul/min constant vs. No Flow	223.1	-86.13 to 532.4	No	ns	0.1545
10ul/min for 5min every 3h vs. No Flow	669.2	463.7 to 874.7	Yes	****	<0.0001
Row 12					
2ul/min constant vs. 10ul/min for 5min every 3h	-419.7	-778.1 to -61.29	Yes	*	0.0232
2ul/min constant vs. No Flow	293.8	-48.54 to 636.2	No	ns	0.0887
10ul/min for 5min every 3h vs. No Flow	713.5	503.0 to 924.1	Yes	****	<0.0001
Row 13					
2ul/min constant vs. 10ul/min for 5min every 3h	-350.7	-721.1 to 19.68	No	ns	0.0632
2ul/min constant vs. No Flow	361.6	3.710 to 719.5	Yes	*	0.0480
10ul/min for 5min every 3h vs. No Flow	712.4	509.1 to 915.6	Yes	****	<0.0001
Row 14					
2ul/min constant vs. 10ul/min for 5min every 3h	-309.3	-711.0 to 92.53	No	ns	0.1338
2ul/min constant vs. No Flow	461.4	68.12 to 854.6	Yes	*	0.0256
10ul/min for 5min every 3h vs. No Flow	770.6	573.3 to 968.0	Yes	****	<0.0001
Row 15					
2ul/min constant vs. 10ul/min for 5min every 3h	-268.0	-683.5 to 147.5	No	ns	0.2221
2ul/min constant vs. No Flow	570.5	155.5 to 985.5	Yes	*	0.0102
10ul/min for 5min every 3h vs. No Flow	838.5	591.9 to 1085	Yes	****	<0.0001
Row 16					
2ul/min constant vs. 10ul/min for 5min every 3h	-235.6	-667.5 to 196.3	No	ns	0.3239
2ul/min constant vs. No Flow	609.6	176.4 to 1043	Yes	**	0.0088
10ul/min for 5min every 3h vs. No Flow	845.2	583.5 to 1107	Yes	****	<0.0001
Row 17					
2ul/min constant vs. 10ul/min for 5min every 3h	-209.0	-618.4 to 200.3	No	ns	0.3629
2ul/min constant vs. No Flow	638.6	233.0 to 1044	Yes	**	0.0053
10ul/min for 5min every 3h vs. No Flow	847.6	639.7 to 1056	Yes	****	<0.0001
Row 18					
2ul/min constant vs. 10ul/min for 5min every 3h	-181.7	-648.5 to 285.1	No	ns	0.5384
2ul/min constant vs. No Flow	652.1	192.4 to 1112	Yes	*	0.0106
10ul/min for 5min every 3h vs. No Flow	833.9	620.6 to 1047	Yes	****	<0.0001
Row 19					
2ul/min constant vs. 10ul/min for 5min every 3h	-148.0	-636.4 to 340.3	No	ns	0.6874
2ul/min constant vs. No Flow	755.1	280.1 to 1230	Yes	**	0.0052
10ul/min for 5min every 3h vs. No Flow	903.1	632.7 to 1174	Yes	****	<0.0001
Row 20					
2ul/min constant vs. 10ul/min for 5min every 3h	-99.54	-591.3 to 392.2	No	ns	0.8380
2ul/min constant vs. No Flow	815.5	330.6 to 1300	Yes	**	0.0040
10ul/min for 5min every 3h vs. No Flow	915.0	677.2 to 1153	Yes	****	<0.0001
Row 21					

2ul/min constant vs. 10ul/min for 5min every 3h	-37.80	-536.4 to 460.8	No	ns	0.9756
2ul/min constant vs. No Flow	892.0	391.2 to 1393	Yes	**	0.0019
10ul/min for 5min every 3h vs. No Flow	929.8	615.9 to 1244	Yes	****	<0.0001
Row 22					
2ul/min constant vs. 10ul/min for 5min every 3h	56.72	-424.3 to 537.8	No	ns	0.9416
2ul/min constant vs. No Flow	961.9	464.5 to 1459	Yes	***	0.0009
10ul/min for 5min every 3h vs. No Flow	905.2	576.9 to 1233	Yes	****	<0.0001
Row 23					
2ul/min constant vs. 10ul/min for 5min every 3h	119.4	-345.2 to 584.1	No	ns	0.7602
2ul/min constant vs. No Flow	1035	559.3 to 1510	Yes	***	0.0004
10ul/min for 5min every 3h vs. No Flow	915.2	595.1 to 1235	Yes	****	<0.0001
Row 24					
2ul/min constant vs. 10ul/min for 5min every 3h	144.7	-317.7 to 607.1	No	ns	0.6713
2ul/min constant vs. No Flow	1127	601.4 to 1653	Yes	***	0.0003
10ul/min for 5min every 3h vs. No Flow	982.4	560.4 to 1404	Yes	***	0.0002
Row 25					
2ul/min constant vs. 10ul/min for 5min every 3h	207.0	-254.0 to 668.0	No	ns	0.4535
2ul/min constant vs. No Flow	1199	658.5 to 1740	Yes	***	0.0002
10ul/min for 5min every 3h vs. No Flow	992.1	550.9 to 1433	Yes	***	0.0004
Row 26					
2ul/min constant vs. 10ul/min for 5min every 3h	205.7	-260.6 to 672.1	No	ns	0.4632
2ul/min constant vs. No Flow	1194	680.3 to 1707	Yes	***	0.0001
10ul/min for 5min every 3h vs. No Flow	988.1	601.5 to 1375	Yes	****	<0.0001
Row 27					
2ul/min constant vs. 10ul/min for 5min every 3h	228.3	-234.5 to 691.2	No	ns	0.3957
2ul/min constant vs. No Flow	1232	730.7 to 1734	Yes	****	<0.0001
10ul/min for 5min every 3h vs. No Flow	1004	618.0 to 1390	Yes	****	<0.0001
Row 28					
2ul/min constant vs. 10ul/min for 5min every 3h	225.2	-265.9 to 716.3	No	ns	0.4413
2ul/min constant vs. No Flow	1285	761.7 to 1808	Yes	****	<0.0001
10ul/min for 5min every 3h vs. No Flow	1060	675.4 to 1444	Yes	****	<0.0001
Row 29					
2ul/min constant vs. 10ul/min for 5min every 3h	258.9	-212.0 to 729.8	No	ns	0.3273
2ul/min constant vs. No Flow	1343	828.8 to 1857	Yes	****	<0.0001
10ul/min for 5min every 3h vs. No Flow	1084	673.5 to 1494	Yes	****	<0.0001
Row 30					
2ul/min constant vs. 10ul/min for 5min every 3h	244.7	-195.1 to 684.4	No	ns	0.3200
2ul/min constant vs. No Flow	1359	873.1 to 1844	Yes	****	<0.0001
10ul/min for 5min every 3h vs. No Flow	1114	719.9 to 1508	Yes	****	<0.0001
Row 31					
2ul/min constant vs. 10ul/min for 5min every 3h	260.5	-166.5 to 687.5	No	ns	0.2656
2ul/min constant vs. No Flow	1389	903.6 to 1875	Yes	****	<0.0001
10ul/min for 5min every 3h vs. No Flow	1129	707.8 to 1550	Yes	****	<0.0001

Row 32					
2ul/min constant vs. 10ul/min for 5min every 3h	229.1	-164.4 to 622.6	No	ns	0.2972
2ul/min constant vs. No Flow	1391	870.9 to 1912	Yes	****	<0.0001
10ul/min for 5min every 3h vs. No Flow	1162	675.6 to 1649	Yes	***	0.0002
Row 33					
2ul/min constant vs. 10ul/min for 5min every 3h	222.3	-147.2 to 591.8	No	ns	0.2723
2ul/min constant vs. No Flow	1348	885.3 to 1811	Yes	****	<0.0001
10ul/min for 5min every 3h vs. No Flow	1126	712.4 to 1540	Yes	****	<0.0001
Row 34					
2ul/min constant vs. 10ul/min for 5min every 3h	197.8	-144.4 to 540.0	No	ns	0.2975
2ul/min constant vs. No Flow	1350	906.5 to 1793	Yes	****	<0.0001
10ul/min for 5min every 3h vs. No Flow	1152	750.4 to 1554	Yes	****	<0.0001
Row 35					
2ul/min constant vs. 10ul/min for 5min every 3h	161.1	-155.1 to 477.3	No	ns	0.3752
2ul/min constant vs. No Flow	1321	875.0 to 1767	Yes	****	<0.0001
10ul/min for 5min every 3h vs. No Flow	1160	750.1 to 1570	Yes	****	<0.0001
Row 36					
2ul/min constant vs. 10ul/min for 5min every 3h	115.4	-194.7 to 425.5	No	ns	0.5854
2ul/min constant vs. No Flow	1319	879.5 to 1758	Yes	****	<0.0001
10ul/min for 5min every 3h vs. No Flow	1203	791.3 to 1615	Yes	****	<0.0001
Row 37					
2ul/min constant vs. 10ul/min for 5min every 3h	86.17	-193.6 to 365.9	No	ns	0.6811
2ul/min constant vs. No Flow	1262	842.8 to 1681	Yes	****	<0.0001
10ul/min for 5min every 3h vs. No Flow	1176	784.1 to 1567	Yes	****	<0.0001
Row 38					
2ul/min constant vs. 10ul/min for 5min every 3h	47.57	-234.4 to 329.6	No	ns	0.8910
2ul/min constant vs. No Flow	1304	847.0 to 1760	Yes	****	<0.0001
10ul/min for 5min every 3h vs. No Flow	1256	817.6 to 1694	Yes	***	0.0001
Row 39					
2ul/min constant vs. 10ul/min for 5min every 3h	43.85	-216.3 to 304.0	No	ns	0.8929
2ul/min constant vs. No Flow	1260	822.4 to 1697	Yes	****	<0.0001
10ul/min for 5min every 3h vs. No Flow	1216	790.7 to 1641	Yes	****	<0.0001
Row 40					
2ul/min constant vs. 10ul/min for 5min every 3h	62.48	-198.3 to 323.2	No	ns	0.7935
2ul/min constant vs. No Flow	1281	811.0 to 1752	Yes	****	<0.0001
10ul/min for 5min every 3h vs. No Flow	1219	761.4 to 1676	Yes	***	0.0002
Row 41					
2ul/min constant vs. 10ul/min for 5min every 3h	46.57	-233.0 to 326.1	No	ns	0.8900
2ul/min constant vs. No Flow	1301	819.0 to 1782	Yes	****	<0.0001
10ul/min for 5min every 3h vs. No Flow	1254	791.4 to 1717	Yes	***	0.0002
Row 42					
2ul/min constant vs. 10ul/min for 5min every 3h	57.62	-232.6 to 347.8	No	ns	0.8462
2ul/min constant vs. No Flow	1233	784.0 to 1682	Yes	****	<0.0001

10ul/min for 5min every 3h vs. No Flow	1175	754.8 to 1596	Yes	***	0.0002
Row 43					
2ul/min constant vs. 10ul/min for 5min every 3h	95.05	-181.9 to 372.0	No	ns	0.6172
2ul/min constant vs. No Flow	1232	798.8 to 1665	Yes	****	<0.0001
10ul/min for 5min every 3h vs. No Flow	1137	730.5 to 1543	Yes	***	0.0002
Row 44					
2ul/min constant vs. 10ul/min for 5min every 3h	70.03	-224.9 to 364.9	No	ns	0.7855
2ul/min constant vs. No Flow	1254	769.9 to 1739	Yes	****	<0.0001
10ul/min for 5min every 3h vs. No Flow	1184	725.5 to 1643	Yes	***	0.0003
Row 45					
2ul/min constant vs. 10ul/min for 5min every 3h	71.43	-222.4 to 365.3	No	ns	0.7745
2ul/min constant vs. No Flow	1253	756.5 to 1749	Yes	***	0.0001
10ul/min for 5min every 3h vs. No Flow	1181	709.0 to 1654	Yes	***	0.0004

Note: * $p < 0.05$;** $p < 0.01$; *** $p < 0.001$; **** $p < 0.0001$. *Statistical significance was assessed using Tukey's multiple comparisons test. Diff. = difference, CI = confidence interval; Sig = Significance; Adj. = adjusted.*
